# Elevated angiopoietin-like protein 3 serum levels in diabetic nephropathy patients and its association with renal function and lipid profile

**DOI:** 10.1186/s12882-023-03214-1

**Published:** 2023-06-13

**Authors:** Samaneh Mohassel Azadi, Reza Fadaei, Ramtin Omid-Shafaat, Jalil Hosseini, Nariman Moradi

**Affiliations:** 1 Department of Clinical Biochemistry, Faculty of Medicine, Tehran University of Medical Sciences, Tehran, Iran; 2 Sleep Disorders Research Center, Kermanshah University of Medical Sciences, Kermanshah, Iran; 3 Student Research Committee, Kurdistan University of Medical Sciences, Sanandaj, Iran; 4 Men’s Health and Reproductive Health Research Center, Shahid Beheshti University of Medical Sciences, Tehran, Iran; 5 Liver and Digestive Research Center, Research Institute for Health Development, Kurdistan University of Medical Sciences, Sanandaj, Iran

**Keywords:** Hepatokine, ANGPTL, Kidney function, Lipoprotein lipase, Cytokine

## Abstract

**Background:**

Type 2 diabetes mellitus (T2DM) is a highly prevalent disease that has life-threatening consequences like micro and macrovascular complication. Diabetic nephropathy (DN) is one of the common consequences of T2DM which is related to secretory factors like hepatokines. Angiopoietin-Like Protein 3 (ANGPTL3) is a hepatokine that is perturbated in cardiometabolic diseases and experimental studies showed its effect on renal functions and lipid metabolism. For the first time, ANGPTL3 was measured in patients with T2DM and DN in the present study.

**Methods:**

Serum levels of ANGPTL3, IL-6, and TNF-α were measured in 60 healthy control, 60 T2DM patients, and 61 DN patients.

**Results:**

Serum levels of ANGPTL3 increased in T2DM (252.39 ± 66.01) and DN (284.59 ± 69.27) patients compared to controls (160.22 ± 48.96), and DN patients compared with T2DM patients. Urinary albumin excretion (UAE) was higher in the DN group compared to T2DM and control groups. Moreover, serum levels of IL-6 and TNF-α were elevated in both patient groups compared to controls. Moreover, ANGPTL3 represented a positive correlation with triglycerides, creatinine, and UAE in patients with both T2DM and DN groups and showed an inverse correlation with eGFR in patients with DN. Moreover, this hepatokine had a good potential to differentiate patients from controls, especially, DN patients.

**Conclusions:**

these findings provide invivo evidence for the relation of ANGPTL3 with renal dysfunction and hypertriglyceridemia in patients with DN which is in line with experimental findings and suggested a potential role for this hepatokine in DN pathogenesis.

## Introduction

In recent decades diabetes mellitus (DM) and its later complications have become a significant health problem worldwide. DM directly relates to metabolic syndrome and an unhealthy lifestyle; therefore, it has a high incidence among people with a sedentary lifestyle and obesity [1]. Microvascular complications of diabetes are retinopathy, neuropathy, and nephropathy. Diabetic nephropathy (DN) is the most common microvascular complication and 30% of kidney transplant recipients suffered from diabetes mellitus. Moreover, DN leads to an increase in the risk of death from cardiovascular diseases [2–5]. Studies have shown that insulin resistance, hyperglycemia, inflammation, oxidative stress, and adipose tissue can play a critical role in the development and progression of DN [6, 7]. Diabetic Nephropathy is associated with the deregulation of adipokines, hepatokines, and myokines such as resistin, adiponectin, leptin, interleukin-6 (IL-6), and other inflammatory markers [8]. Myokine and hepatokine regulate pathophysiological processes in the body like insulin sensitivity, inflammation, and lipid metabolism [9]. For example, fetuin-A as a potent vascular calcification inhibitor was found to be inversely associated with all causes of mortality in patients with chronic kidney disease (CKD) [10]. Furthermore, fibroblast growth factor 21 (FGF21) plays an important role in lipid and glucose metabolism and its level increased in patients with CKD [11].

Angiopoietin-like proteins (ANGPTLs) as a family of hepatokine are involved in some critical processes in the body like lipid and glucose metabolism. This family consists of 8 proteins that have structural similarities to the angiopoietin protein “vascular endothelial growth factors’’ family [12] with potential functional diversity properties due to their different receptor binding [13]. One of the most researched roles of ANGPTLs is their activity in lipid metabolism, specially ANGPLT3 and ANGPTL4 [14]. ANGPTL4 acts as an inhibitor of lipoproteins lipase (LPL) which suppresses triglycerides clearance from circulation [15]. ANGPTL3 is a 460 amino acids glycoprotein [12, 16] that is mainly produced in hepatocytes and kidney podocytes and, as a hepatokine, plays a crucial role in lipid metabolism and glucose hemostasis [17]. ANGPTL3 acts as an inhibitor of LPL and endothelial lipase activity and regulates triglycerides and lipoprotein metabolism [18].

Interestingly, the potential role of ANGPTL3 and other hepatokines and adipokines in T2DM and metabolic syndromes is the point of interest in several studies. Cinkajzlová et al. studied serum levels of ANGPTL3 in patients with T2DM and obesity which showed different levels in patients and controls [19]. Another study investigated levels of ANGPTL3 in patients with primary nephrotic syndrome, and ANGPTL3 was found to be elevated in patients and correlated with lipid and lipoprotein profiles [20]. Moreover, there is evidence for the influence of ANGPTL3 on renal function and kidney structure [21, 22], but to our knowledge, there is no data on serum levels of ANGPTL3 in patients with diabetic nephropathy. Therefore, this study sought to investigate levels of ANGPTL3 in serum samples of DN patients and its relation with lipid metabolism.

## Method

### Study population

This case-control study included 60 patients with type 2 diabetes mellitus (T2DM), 61 patients with diabetic nephropathy (DN), and 60 controls. T2DM was diagnosed according to the criteria of the American Diabetes Association. All outpatients were referred to Shohada Tajrish Hospital and Institute of Endocrinology and Metabolism, Tehran, Iran from January 2019 to January 2020. Diagnosis of DN was based on urinary albumin excretion (UEA) level, and a UAE > 200 µg / min, confirmed through repeat measurements taken at least 3 to 6 months apart, is considered indicative of DN.

The study excluded individuals with a prior history or current evidence of cancer, autoimmune diseases, type 1 diabetes, infectious diseases, as well as other kidney diseases such as nephrotic syndrome, urinary tract infections, and nephrolithiasis. All participants provided written consent before taking part in the study. The study was performed according to the Declaration of Helsinki and approved by the Ethics Committee of Shahid Beheshti University of Medical Sciences (IR.SBMU.RETECH.REC.1398.432).

### Anthropometric data and biochemical measurements

Height and weight were measured to calculate body mass index (BMI) and standard barometers were used to determine systolic blood pressure (SBP) and diastolic blood pressure (DBP). Five milliliters (mL) were taken from all participants after overnight fasting. Furthermore, fasting blood sugar (FBS), lipid profiles including total cholesterol (TC), triglyceride (TG), low-density cholesterol (LDL-C) and high-density lipoprotein (HDL-C) as well as Creatinine (Cr), UAE, alanine aminotransferase (ALT) and aspartate aminotransferase (AST) were measured using commercial kits (Parsazmon, Iran).

Traditional 4 variables the diet modification equation in kidney disease (MDRD) was used to calculate the estimated glomerular filtration rate (eGFR). Insulin was measured with ELISA kit (Monobind, USA) and HOMA-IR calculated with standard formula: FBS (mg / dL) / insulin (uU/mL) 405).

Measuring serum ANGPTL3 and cytokines.

Serum ANGPTL3 levels were determined by ELISA kit (Aviscera Bioscience, Inc., USA), with intra- and inter-assay coefficients of variants (CV) of 6% and 9%, respectively. Tumor necrosis factor-α (TNF-α) and interleukin-6 (IL-6) were assessed by ELISA kit (R & D Systems USA) with a minimum detectable dose of 1.6 and 0.7 pg/mL, respectively.

### Statistical analysis

All statistical analyzes were performed using SPSS software version 16. Categorical data are shown by frequency and percentage and tested using chi-square. Continuous data are presented by the mean and standard deviation (SD) and tested using the student t-test or one-way ANOVA. Pearson correlation test was applied to test the correlation of ANGPTL with other variables. In addition, binary logistic regression was carried out to find an odd ratio of disease status according to ANGPTL and three models were applied for adjustment of confounding factors; Model-1 included adjustments for age, sex, and BMI. Model-2 included adjustments for TG and Model-1 variables, while Model-3 included adjustments for variables in Model-2 as well as IL-6 and TNF-α. The ROC curve was plotted to test the diagnostic ability of ANGPTL3. P value less than 0.05 is considered a significant threshold.

## Results

### Studied population

The basic characteristics of the studied population are given in Table 1. Patients and controls showed no considerable change in terms of age and BMI. While SBP was higher in DN compared to T2DM and control groups, SBP was higher in DN and T2DM groups compared to controls. As expected FBG, insulin, HOMA-IR, and HbA1c indicated a higher concentration in both patient groups compared to controls, and insulin, HOMA-IR, and HbA1c were considerably elevated in the DN group compared to T2DM. TG levels were found to be higher in patients with T2DM and DN compared to controls and also in DN compared to the T2DM group. TC and LDL-C levels were found to be higher concentration in DN compared to controls and LDL-C represented an elevated level in DN compared to T2DM. patients with DN and T2DM showed a lower HDL-C concentration compared to controls. Cr and UAE were found to be higher in the DN group compared to T2DM and control groups.

**Table 1:** Basic characteristics of the studied population

Variables	Control (n = 60)	T2DM (n = 60)	DN (n = 60)	P value
Age (year)	60.1 ± 9.77	60.32 ± 9.56	61.72 ± 9.38	0.600
BMI (kg/m^2^)	24.71 ± 3.30	24.83 ± 3.86	25.97 ± 3.92	0.122
SBP (mmHg)	131.85 ± 14.22	136.43 ± 18.51	146.36 ± 19.62 ^b**, c**^	< 0.001
DBP (mmHg)	84.47 ± 13.58	91.47 ± 18.64^a*^	95.25 ± 13.96 ^b**^	0.001
FBG (mg/dl)	88.74 ± 7.67	157.04 ± 18.51 ^a**^	161.16 ± 18.19 ^b**^	< 0.001
Insulin (uU/mL)	3.10 ± 1.78	10.07 ± 3.55 ^a**^	13.06 ± 5.12 ^b**, c**^	< 0.001
HbA1c (%)	3.51 ± 0.97	8.24 ± 1.61^a**^	9.53 ± 1.60 ^b**, c**^	< 0.001
HOMA-IR	0.68 ± 0.4	3.93 ± 1.53 ^a**^	5.22 ± 2.21 ^b**, c**^	< 0.001
TG (mg/dL)	126.85 ± 47.55	151.42 ± 41.08 ^a**^	175.13 ± 57.85 ^b**, c**^	< 0.001
TC (mg/dL)	173.34 ± 38.71	182.62 ± 45.99	195.36 ± 46.46 ^b**^	0.023
LDL-C (mg/dL)	106.10 ± 31.14	107.97 ± 34.81	121.05 ± 34.14 ^b**, c*^	0.029
HDL-C (mg/dL)	43.42 ± 6.59	38.11 ± 7.08 ^a**^	39.64 ± 5.64 ^b**^	< 0.001
Cr (mg/dL)	1.20 ± 0.17	1.24 ± 0.12	2.67 ± 0.79 ^b**, c**^	< 0.001
UAE (µg/min)	11.56 ± 4.44	12.69 ± 5.67	266.67 ± 74.24 ^b**, c**^	< 0.001
eGFR	61.53 ± 14.42	57.63 ± 10.75	26.08 ± 9.94 ^b**, c**^	< 0.001

### Serum levels of cytokines and ANGPTL3

**Fig. 1:**
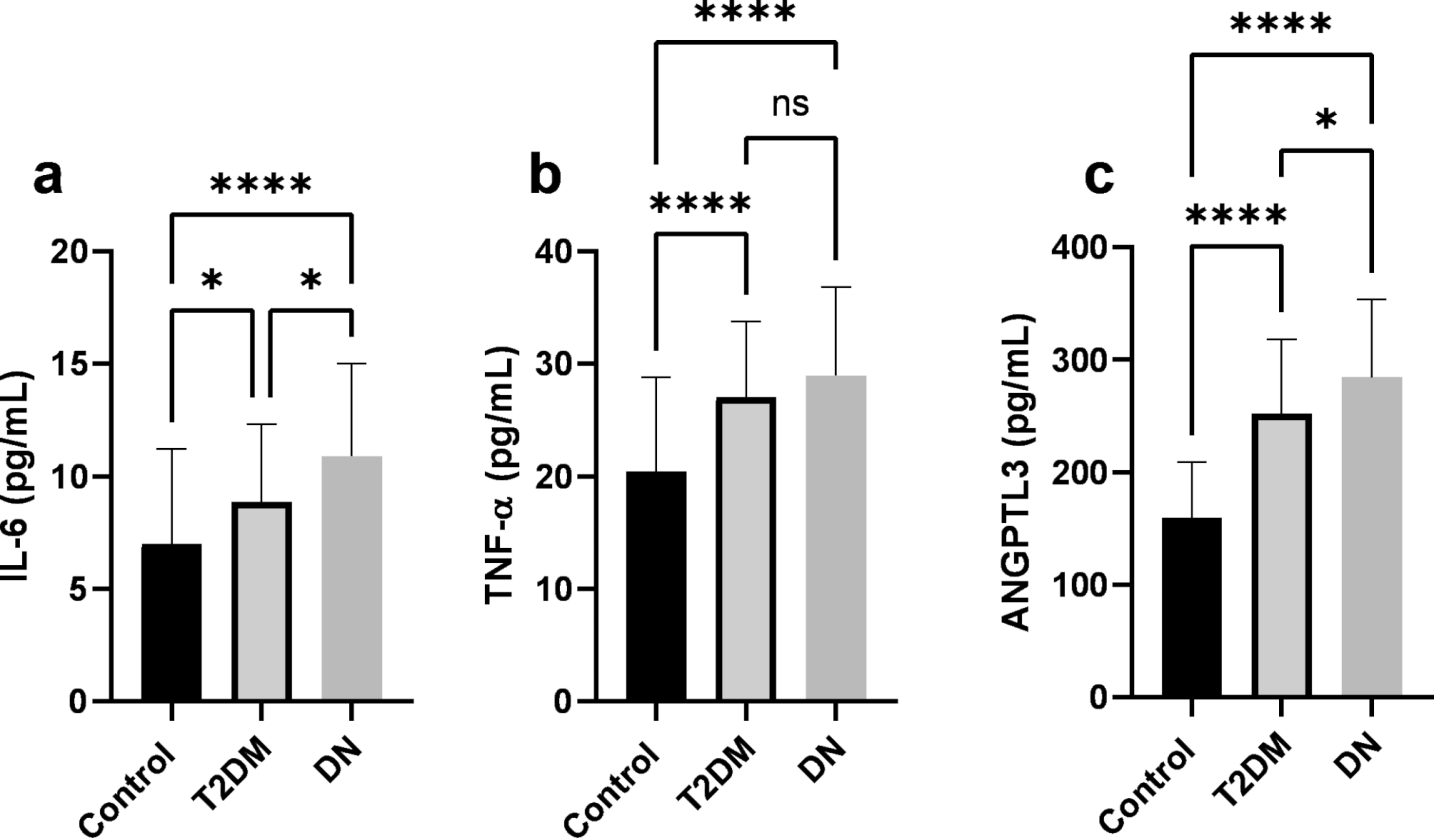
**A**) serum levels of IL-6 elevated in patients with DN compared to T2DM and control groups and DN patients compared to T2DM patients. **B**) Serum levels of TNF-α were found to be higher in both patient groups compared to controls. **C**) Levels of ANGPTL3 a higher concentration in T2DM and DN groups in comparison to control and in patients with DN compared to patients with T2DM. T2DM, type 2 diabetes mellitus; DN, diabetic nephropathy. * p < 0.05, **** p < 0.0001

Serum levels of IL-6 indicated a higher concentration in DN (10.91 ± 4.09) and T2DM (8.88 ± 3.45) groups compared to controls (7.01 ± 4.21), and in DN compared to the T2DM group (Fig. 1a). TNF-α showed a higher concentration in DN (28.98 ± 7.84) and T2DM (27.05 ± 6.74) groups compared to controls (20.45 ± 8.37) (Fig. 1b).

ANGPLT3 serum concentration was elevated in patients with DN (284.59 ± 69.27) and T2DM (252.39 ± 66.01) compared to control (160.22 ± 48.96), and in patients with DN compared to T2DM (Fig. 1c). ANCOVA was performed to eliminate the impact of the covariates (sex, age, and BMI) on serum levels of ANGPTL3 and the results remained unchanged.

In addition, multinomial logistic regression was performed to assess the odd ratio of disease status according to serum levels of ANGPTL3, and the results are shown in Table 2. ANGPTL3 represented a significant association with risk of disease status and the results remained unchanged after adjustment for age, sex and BMI, TG and inflammatory markers.

**Table 2:** The odd ratio of disease status according to 10 unit change in the serum levels of ANGPTL3.

Group		B	Std. Error	Wald	Odd ratio (B)	95% Confidence Interval for Exp(B)		P value
						Lower Bound	Upper Bound	
T2DMs	crude	0.265	0.046	33.083	1.304	1.191	1.427	< 0.001
	adjusted	0.285	0.049	33.930	1.329	1.208	1.463	< 0.001
	Adjusted model-2	0.281	0.05	31.184	1.325	1.200	1.462	< 0.001
	Adjusted model-3	0.274	0.052	27.901	1.315	1.188	1.456	< 0.001
DN	Crude	0.337	0.050	46.052	1.401	1.271	1.544	< 0.001
	Adjusted model-1	0.347	0.052	44.294	1.416	1.278	1.568	< 0.001
	Adjusted model-2	0.332	0.054	38.405	1.394	1.255	1.549	< 0.001
	Adjusted model-3	0.342	0.056	37.340	1.408	1.261	1.571	< 0.001

The adjustment was performed for the following factors in each model:

Model-1: age, sex, and BMI; Model-2: age, sex, BMI and TG; Model-3: age, sex, BMI, TG, TNF-α and IL-6.

**Fig. 2:**
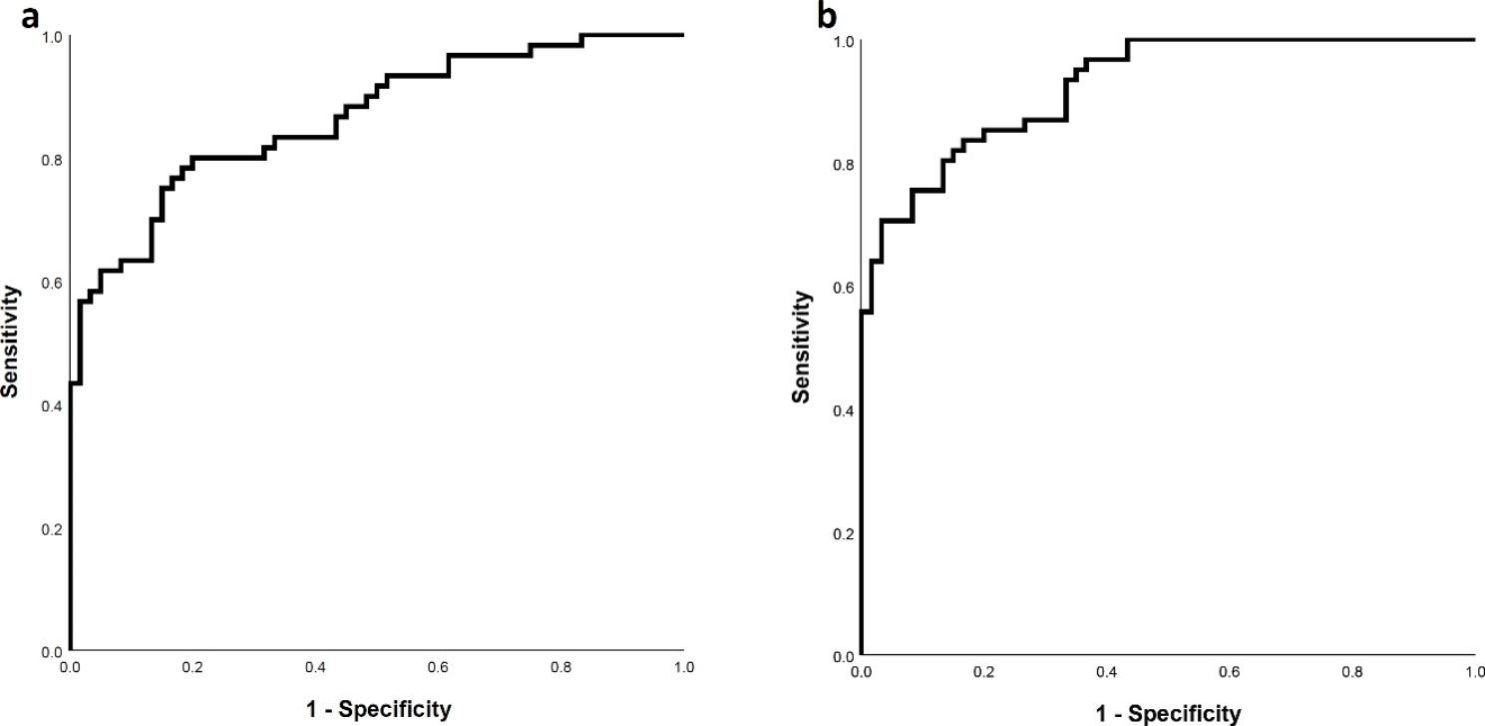
ROC curve analysis to test the diagnostic ability of ANGPTL3 to differentiate controls from (**A**) type 2 diabetes mellitus, and (**B**) diabetic nephropathy

The ability of ANGPTL3 to differentiate diseases status from control were evaluate using ROC curve analysis and the result represented a good ability for ANGPTL3 to distinguish between control and T2DM (cut-off: 202.01 pg/mL, area under curve (95% CI): 0.864 (0.800, 0.928), p < 0.001) (Fig. 2a) and DN (cut-off: 201.95 pg/mL, area under curve (95% CI): 0.925 (0.882, 0.968), p < 0.001) (Fig. 2b).

### Relation of ANGPTL3 with other variables

Serum levels of ANGPTL3 were tested according to sex and the results showed no considerable change between males (237.5 ± 81.83) and females (224.69 ± 80.32, p = 0.304).

Correlation analysis was performed in each group separately and the results are given in Table 3. In the control group, ANGPTL3 positively correlated with TG. In both patient groups, ANGPTL3 positively correlated with BMI, TG, Cr, and UAE. Moreover, ANGPTL3 is inversely correlated with eGFR in patients with DN.

**Table 3:** Pearson correlation of ANGPTL3 with other variables

	Control	T2DM	DN
Age	0.045	0.201	0.061
BMI	0.096	0.283*	0.271*
SBP	-0.032	-0.051	0.056
DBP	-0.055	-0.087	0.057
HbA1c	0.307^*^	-0.088	0.125
FBG	0.216	0.014	0.011
Insulin	0.109	0.162	0.081
HOMA-IR	0.143	0.145	0.079
TG	0.457^**^	0.418**	0.425**
TC	0.045	0.220	0.115
LDL-C	0.042	0.211	0.069
HDL-C	-0.189	-0.088	0.097
Creatinine	0.191	0.296*	0.372*
UAE	-0.116	0.295*	0.353*
eGFR	-0.095	-0.064	-0.269*
IL-6	-0.062	0.098	0.074
TNF-α	-0.125	0.067	-0.072

## Discussion

The main finding of the present study is elevated levels of ANGPT3 in DN and T2DM patients compared to controls and its relation with parameters of kidney function and lipoprotein metabolism. This is the first report on serum levels of ANGPTL3 in patients with DN, while there are studies that reported ANGPTL3 in diabetic retinopathy and primary nephrotic syndrome.

In line with the present study, Zhong et al. reported elevated levels of ANGPTL3 in patients with primary nephrotic syndrome, and another study showed an independent relation of ANGPTL3 with retinopathy [20, 23]. Strikingly, ANGPTL3 was found to be associated with proteinuria in hyperlipidemic patients [24]. In addition, there is evidence of change in circulating ANGPTL3 in patients with diabetes mellitus. Animal model for diabetes mellitus represent a higher concentration of ANGPTL3 and patients with T2DM represent similar status for this hepatokine [25, 26], however, Zhao et al. demonstrated lower levels of ANGPTL3 in the female with T2DM compared to controls and no change between men with T2DM and control [27], this controversy might be a result from medications and difference in ethnic.

Our findings showed a relation of ANGPTL3 with the marker of kidney function, especially in patients with DN. There are several lines of evidence that can describe the underlying mechanism for this relation. Injuries induced by puromycin-induced in cultured podocytes are followed by upregulation of ANGPTL3 which is associated with reduction of agrin and perlecan expression [28]. Moreover, ANGPTL3 knockdown attenuated podocyte injury and proteinuria induced by Adriamycin [21]. Similar results were found in another study which demonstrated a protective role for ANGPTL3 knockdown against structural changes and kidney dysfunction [22]. These findings showed that ANGPTL3 plays an unfavorable role in kidney function and the results of the present study is in vivo evidence for the relation of ANGPTL3 with renal dysfunction in patients with DN.

In addition to the relation of ANGTPL3 with renal dysfunction, this hepatokine demonstrated a positive relationship with TG levels which is in line with previous findings. There are several lines of evidence that ANGPTL3 is associated with lipid profile and lipoprotein metabolism [29, 30], ANGPTL3 indicated a positive association with TG in the Finnish population [31], patients with polycystic ovary syndrome [17] preeclamptic pregnant women [32]. Interestingly, ANGPTL3 showed a relation with proteinuria in patients with hyperlipidemia [24]. Experimental studies prove the inhibitory impact of ANGPTL3 on LPL [33]. ANGPTL3 loss of function mutation leads to a disorder called familial combined hypobetalipoproteinemia [34], and cleavage of ANGPTL3 by ANGPTL8 enhances the inhibitory impact of ANGTPL3 on LPL activity that results in an elevation in the levels of TG [35, 36]. These mechanisms might be the underlying cause for the relation of ANGPTL3 with TG levels in the present study.

Collectively, the present study showed an in vivo relation of ANGPTL3 with markers of renal dysfunction and hypertriglyceridemia in patients with DN, which is in line with previous clinical and experimental studies and these findings suggested ANGPTL3 as a potential therapeutic target for renal dysfunction in patients with diabetes, although further interventional studies are required in this regard.

Strength and limitations: This study was performed on a population with well-defined inclusion and exclusion criteria and matched in terms of age, sex and BMI. Although there are some limitations for the present study; nephropathy was not confirmed by the kidney biopsy and the cross-sectional design of the study limited us to concluding a causal relation between ANGPTL3 and renal function.

## Data Availability

The data that support the findings of this study are available on request from the corresponding author.
